# Electronic cigarette menthol flavoring is associated with increased inhaled micro and sub-micron particles and worse lung function in combustion cigarette smokers

**DOI:** 10.1186/s12931-023-02410-9

**Published:** 2023-04-11

**Authors:** Divay Chandra, Rachel F. Bogdanoff, Russell P. Bowler, Kambez H. Benam

**Affiliations:** 1 Division of Pulmonary, Allergy and Critical Care Medicine, Department of Medicine, University of Pittsburgh, Pittsburgh, PA 15213 USA; 2 Division of Pulmonary, Critical Care and Sleep Medicine, National Jewish Health, Denver, CO 80206 USA; 3 Department of Bioengineering, University of Pittsburgh, Pittsburgh, PA 15219 USA; 4 Vascular Medicine Institute, University of Pittsburgh, Pittsburgh, PA 15213 USA

**Keywords:** Menthol, Electronic cigarette, Pulmonary toxicity, Lung function, Robotic human vaping mimetic real-time particle analyzer, HUMITIPAA

## Abstract

Flavored electronic cigarettes (ECs) present a serious health challenge globally. Currently, it is unknown whether the addition of highly popular menthol flavoring to e-liquid is associated with changes in the number of aerosolized particles generated or altered lung function. Here, we first performed preclinical studies using our novel robotic platform Human Vaping Mimetic Real-Time Particle Analyzer (HUMITIPAA). HUMITIPAA generates fresh aerosols for any desired EC in a very controlled and user-definable manner and utilizes an optical sensing system to quantitate and analyze sub-micron and microparticles from every puff over the course of vaping session in real-time while emulating clinically relevant breathing mechanics and vaping topography. We discovered that addition of menthol flavoring to freshly prepared e-liquid base propylene glycol–vegetable glycerin leads to enhanced particle counts in all tested size fractions, similar to the effect of adding vitamin E acetate to e-liquid we previously reported. Similarly, we found that menthol vs. non-menthol (tobacco) flavored pods from commercially available ECs leads to generation of significantly higher quantities of 1–10 µm particles upon inhalation. We then retrospectively analyzed data from the COPDGene study and identified an association between the use of menthol flavored ECs and reduced FEV1% predicted and FEV1/FVC independent of age, gender, race, pack-years of smoking, and use of nicotine or cannabis-containing vaping products. Our results reveal an association between enhanced inhaled particle due to menthol addition to ECs and worse lung function indices. Detailed causal relation remains to be demonstrated in future large-scale prospective clinical studies. Importantly, here we demonstrate utility of the HUMITIPAA as a predictive enabling technology to identify inhalation toxicological potential of emerging ECs as the chemical formulation of e-liquid gets modified.

**Supplementary Information:**

The online version contains supplementary material available at 10.1186/s12931-023-02410-9.

## Background

A steep increase in the use of electronic cigarettes (ECs) necessitates better understanding of their potential to cause harm. The majority of EC-users consume flavored products [1], and menthol continues to be a highly popular and controversial flavor [2–5]. In fact, there is growing interest in further regulating menthol-flavored ECs to benefit public health, particularly to reduce addiction and youth experimentation [4]. To date a number of in vitro and in vivo studies have been performed on toxicological potential of EC menthol flavoring [6–11]. However, these works mostly used e-liquid, instead of aerosolized EC vapor from menthol-containing e-liquid, to directly challenge pulmonary and non-pulmonary cells in vitro or ex vivo [6, 10], or utilized immortalized cell lines rather than primary human-derived cells [8, 9, 11]. In addition, to our knowledge, no single clinical study has directly evaluated impact of menthol flavoring in ECs on pulmonary function indices. As such, new experimental and clinical evidence on whether the addition of menthol flavoring to ECs can lead to pulmonary toxicity would be highly desirable.

Vitamin E acetate (VEA), a dietary compound used as a diluent in some cannabis-containing ECs and vaping products, has been strongly linked with the EC, or Vaping, Product Use-associated Lung Injury (EVALI) outbreak [12–14]. EVALI unfortunately led to considerable morbidity and mortality. Thus far, the specific cause(s) of EVALI is unknown; however, the analyses of products and patient samples have implicated VEA [12, 14]. Consequently, to learn whether VEA impacts the profile (size distribution and quantity) of inhaled particles from ECs, we recently designed and developed a first-in-kind biologically inspired robotic system (hereon referred to as Human Vaping Mimetic Real-Time Particle Analyzer [HUMITIPAA] [15–17]) that generates fresh aerosols for any desired EC in a controlled and user-definable manner and utilizes an optical sensing system to quantitate and analyze sub-micron and microparticles (300 nm–10 µm) from every puff over the course of vaping session in real-time. Applying HUMITIPAA we revealed that addition of even very small quantities of VEA to e-liquid leads to significantly enhanced particle counts entering the lungs and alters their size distribution [16].

Here, our primary objective was to demonstrate utility of HUMITIPAA as an enabling technology that supports reliable and rapid prediction of pulmonary toxicity potential of emerging ECs after it has been validated in a side-by-side preclinical-clinical association analysis. We focused on menthol flavoring as test article of concern. Currently, there is a pressing gap for such biomimetic systems as many new vaping products with diverse chemical compositions enter the market daily. Existing state-of-the-art preclinical lung models such as air–liquid interface (ALI) culture of human airway epithelial cells (hAEpCs) in transwell inserts (TWIs) or precision-cut lung slices cannot be readily scaled for rapid on-the-fly toxicity testing of the newly emerged ECs and exposure in these models often occurs by submerging the cells under e-liquid, which is not physiological as natural ALI is lost. Therefore, HUMITIPAA, while acellular, can provide the scientific and regulatory community with a more appropriate preclinical tool for inhalation toxicology of ECs.

Increased particles generated from an EC can correlate with enhanced deposition in the respiratory tree and therefore augment likelihood of pulmonary toxicity. In this study, we hypothesized that addition of menthol flavoring to ECs leads to enhanced particle count and that such increase would be associated with worse clinical outcome. Thus, we first utilized HUMITIPAA to evaluate impact of menthol flavor addition to e-liquid base propylene glycol (PG): vegetable glycerin (VG) on physical characteristics of generated particles. Next, we analyzed inhaled particles from two popular and commercially available ECs, one containing menthol and the other containing a non-menthol (tobacco) flavoring (control). Lastly, since no large-scale menthol-flavored EC-focused clinical study exists, next we performed a retrospective analysis of the COPDGene study to complement our preclinical findings.

## Materials and methods

### Sample preparation and inhaled particle physical characterization

**Fig. 1:**
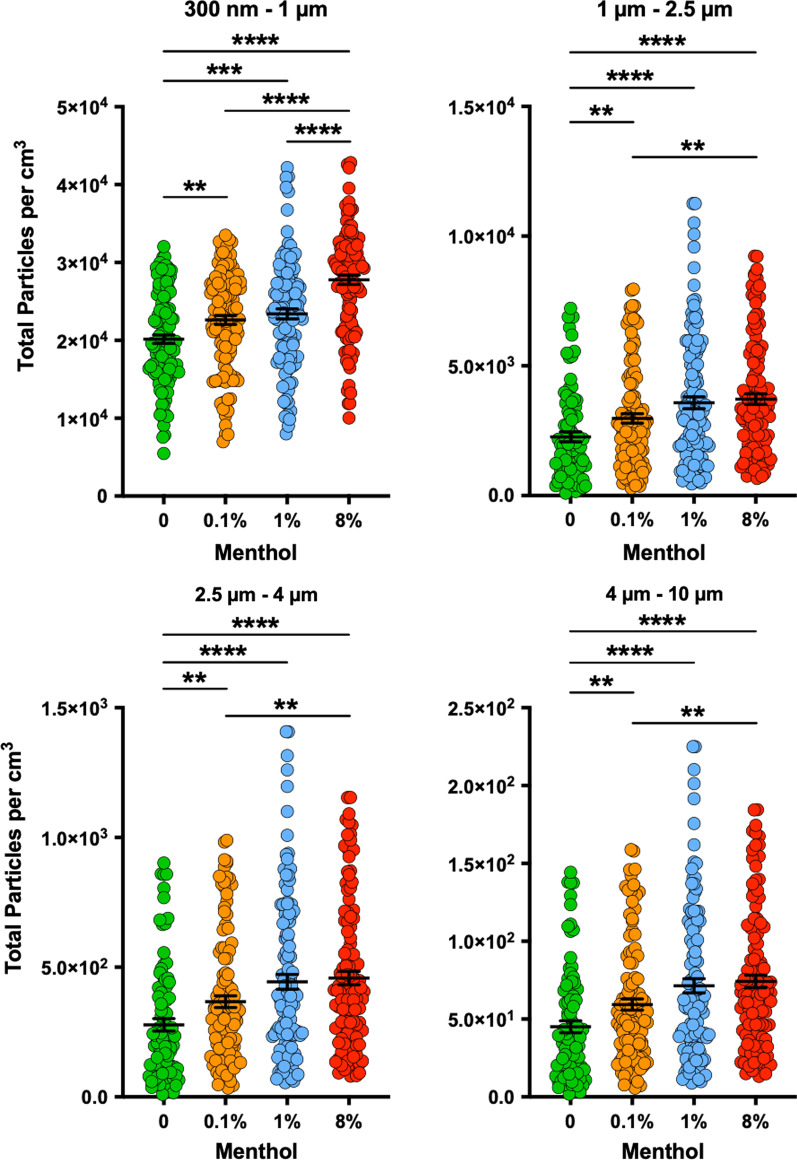
Impact of PG:VG supplemented with menthol flavoring on inhaled particle count. Distribution profiles of the real-time particle count per cm^3^ by HUMITIPAA spanning twenty 5.9-s breathing intervals (6 samples per interval) for each size fraction (300 nm–1 μm, 1 μm–2.5 μm, 2.5 μm–4 μm, 4 μm–10 μm) using a base e-liquid mixture of 50:50 (v/v) PG:VG containing various menthol concentrations. *Mann–Whitney U* tests revealed statistical significance. **P < 0.01, ***P < 0.001, ****P < 0.0001. Results depicted are obtained from 4 independent trials with 5 puffs per trial per condition. Each data point represents the real-time HUMITIPAA Particulate Matter sensor reading that is taken approximately every second during the sampling peak. *HUMITIPAA* Human Vaping Mimetic Real-Time Particle Analyzer, *PG* Propylene Glycol, *VG* Vegetable Glycerin

**Fig. 2:**
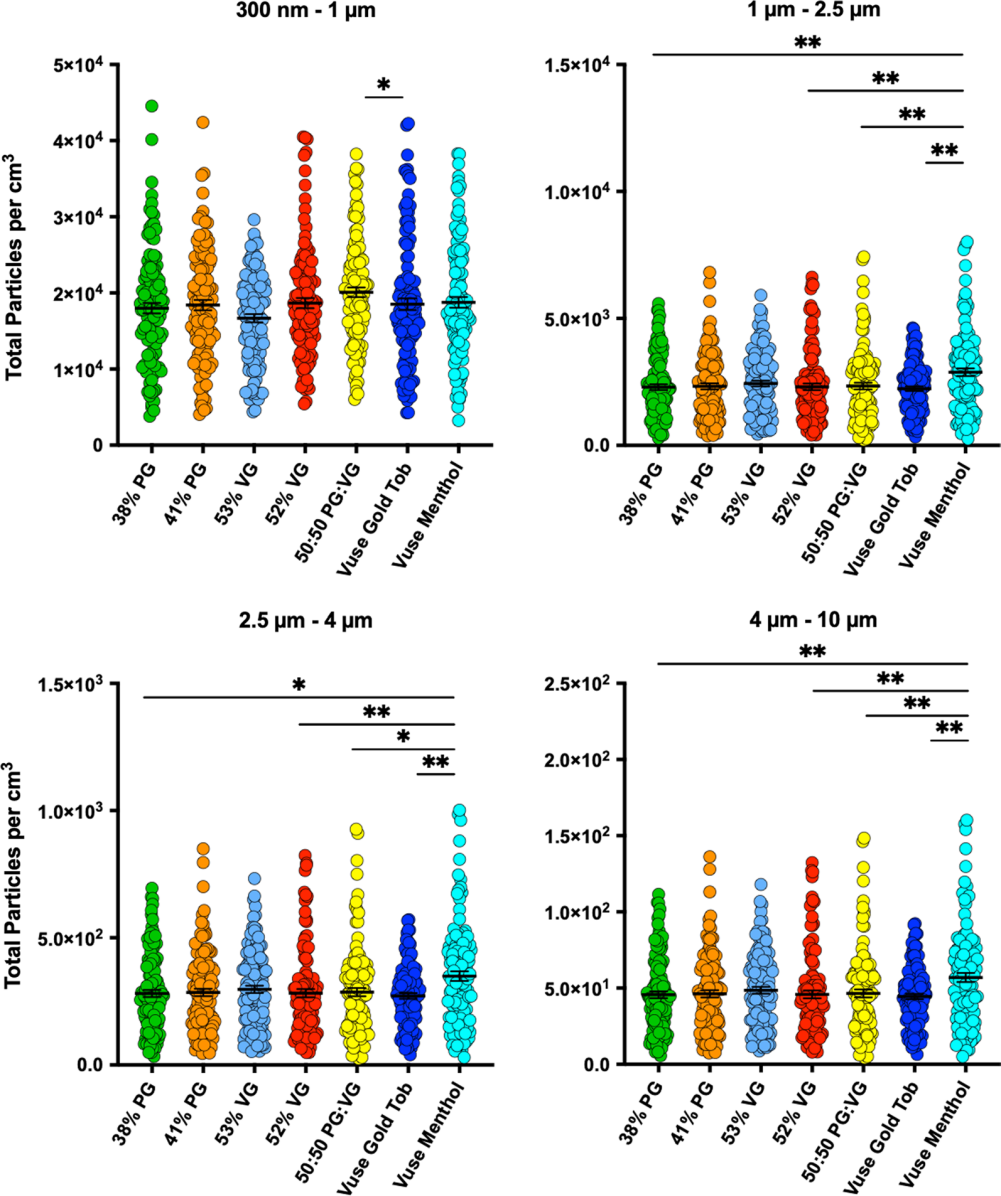
Impact of Menthol Flavoring in Commercially Available Electronic Cigarettes on Inhaled Particle Quantity. Inhaled particle count analysis was performed as in Fig. 1 using HUMITIPAA on popular and off-the-shelf products Vuse Alto Menthol (1.8% nicotine strength) and Vuse Alto Golden Tobacco (1.8% Nicotine) pods, with latter serving as control flavoring. These e-liquids were analyzed by GC–MS (see Table 2) and were found to have PG:VG ratios of 38:52 (Menthol) and 41:53 (Golden Tobacco); hence, we studied particle quantities for all size fractions compared with these PG and VG values in addition to reference 50%. *Mann–Whitney U* tests was applied for statistical significance analysis between the 50:50 PG:VG, 38% PG and 52% VG solutions against the Vuse Alto Menthol, the 50:50 PG:VG, 41% PG and 53% VG solutions against the Vuse Alto Golden Tobacco, and the Vuse Alto Menthol vs. Vuse Alto Golden Tobacco. *P < 0.05, **P < 0.01. Note significantly higher inhaled particles in menthol- vs. non-menthol-flavored EC (4 independent trials, 5 puffs per trial, each data point representing real-time reading by HUMITIPAA sensor at about every second). Electronic Cigarette; *GC–MS* gas chromatography–mass spectroscopy, *Tob* tobacco

The particle characterization was performed using HUMITIPAA robotic system, which we had recently developed and tested [16, 18]. For studies in Fig. 1, we first prepared an e-liquid base by adding equal volumes of PG (Sigma-Aldrich, Cat. #: W294004-1KG-K) to VG (Sigma-Aldrich, Cat. #: W252506-1KG-K), followed by their thorough mixing using a planetary centrifugal mixer (Thinky, Model #: ARE-310). Next, we prepared 8% (wt./v) solution of menthol (Sigma-Aldrich, Cat. #: M2772) in this 50:50 (v/v) PG:VG mixture, and subsequent menthol solutions were created by mixing the 8% menthol solution with the 50:50 PG:VG. For studies in Fig. 2, the control solutions with varying PG and VG levels were created by first preparing a base 50:50 PG:VG mixture as described above; and then incorporating VG to achieve desired ratios. These mixtures contained both PG and VG but were prepared so that one component (PG or VG) at a time matched the corresponding abundance level in commercial e-liquid. No water or other solution/material was used. The quantities of the base mixture and VG used to create each PG:VG ratios for studies in Fig. 2 are summarized in Additional file 1: Table S1. When operating HUMITIPAA, vaping topography parameters mimicked the 2018 International Organization for Standardization (ISO) definitions and standard conditions for vapor production were: puff volume: 55 mL, puff duration: 3 s, and puff profile (ISO 20768:2018) [19]. The breathing behavior of a healthy human adult with a representative respiration cycle of 5.9 s were reproduced. To ensure real-time particle quantification falls within the detection limits of the sensor in our robotic system, we evaluated various dilutions and chose an aerosol dilution factor appropriate for our studies. The Vaping Robot in HUMITIPAA used an EC which contained a heating element to produce the aerosols. The EC used throughout the experiments was a Vaporesso XROS Mini. This device has interchangeable pods which hold the e-liquid and contain the heating coil. The resistance used for the heating coils was 1.2 Ohm and the device wattage was 16 W. The only commercial products used in the HUMITIPAA for studies here were Vuse Alto Menthol and Vuse Alto Golden Tobacco ECs. For this, the e-liquid was removed from the Vuse pods and used with the same device that was used for the other conditions. The air used for dilution was filtered ambient air from the Inhalation Exposure Chamber, and as such it was at 37 °C and 70% relative humidity (the maximum humidity level we can currently achieve without inadvertently damaging the electronics and some mechanical components of the HUMITIPAA).

### Gas chromatography–mass spectroscopy

The gas chromatography–mass spectroscopy (GC–MS) analysis (Table 1a, b) was performed by RJ Lee Group (Monroeville PA, USA). In brief, 10 µL of e-liquid was diluted in 1 mL of methanol. 1 µL of the diluted sample was then loaded into an Agilent sampling vial. Using an Agilent Autosampler, the solution was then injected into the injection port of an Agilent 6890 Gas Chromatograph equipped with a 5973 Mass Selective mass spectrometer. The instrument was run by Mass Hunter Software version 10.1.49 copyright 2020. The data package utilized software containing the latest version, 2021, of a NIST/Wiley library package. The software was utilized to integrate and identify the peaks.

**Table 1:** Menthol abundance in commercially available e-liquids as analyzed by gas chromatography–mass spectroscopy

Commercial e-liquid	Menthol abundance (%)
Vuse pod menthol, 1.8% nicotine	3.8
Vuse pod menthol, 5% nicotine	3.4
Juul pod menthol, 3% nicotine	3.7
Blu polar mint, 2.4% nicotine	3.4
Blu menthol, 2.4% nicotine	0.4
Njoy pod menthol, 2.4% nicotine	1.0
Meta drop cool mint, 5% nicotine	4.1

### Patient cohort

Enrollment criteria for the COPDGene cohort have been described previously [20, 21]. In brief, participants were 45–80 years old with > 10 pack-years of current or prior smoking and without prior thoracic surgery or lung disease besides asthma or COPD. The presence of airflow obstruction, i.e., FEV_1_/FVC < 0.70, was not required for enrollment. Therefore, our study included smokers with and without spirometry airflow obstruction. The Institutional Review Boards at all participating sites approved the study. Written informed consent was obtained from each participant.

During the 5-year and 10-year follow-up visits, EC questionnaires were administered. These included detailed questions on the duration and use of various EC products and flavorings. We analyzed data from the 10-year follow-up visit as the duration of EC exposure was short at the 5-year visit (median of four months, IQR of 12.8 months at five years vs. median of 12 months, IQR of 22 months, at the 10-year visit).

Post-bronchodilator spirometry was performed and adjusted to standard population-derived predicted values [22]. CT images were used to assess emphysema by density mask analysis (LAA% − 950) and gas trapping as described previously [20]. Respiratory symptoms were assessed using the MMRC dyspnea score and the St. George's Respiratory Questionnaire.

### Statistical analysis

#### Preclinical study

The particle counts were analyzed by non-parametric two-tailed *Mann–Whitney U* test. Differences were considered statistically significant when *P* < 0.05 (*P < 0.05, **P < 0.01, ***P < 0.001, ****P < 0.0001). Analyses were performed using GraphPad Prism version 9.4.1.

#### Clinical study

First, the characteristics of menthol users were compared to those using other flavored products. Users of other flavored products were chosen as the comparison group with the expectation that they would be most similar to users of menthol flavoring. Continuous variables were summarized as mean ± standard deviation if normally distributed and median with inter-quartile range (IQR) if not normally distributed. Categorical variables were compared using the Chi^2^ test, normally distributed continuous variables were compared using the t-test, and non-normally distributed continuous variables were compared using the *Kruskal–Wallis* test.

Next, we performed multivariable regression modeling to examine the association between the use of menthol-flavored ECs and lung function. These models were adjusted for age, gender, race, pack-years of smoking, and use of vaping products containing nicotine and cannabis.

Analyses were performed using Stata MP version 17 (StataCorp, College Station, TX, USA). Statistical significance was defined as two-tailed *P* < 0.05.

## Results

In preclinical studies, we set the HUMITIPAA to recreate a puff volume of 55 mL and puff duration of 3 s for vaping topography based on ISO 20768:2018 [19] and emulate normal healthy rhythmic breathing. This allowed us to study inhaled particle profile in a representative non-diseased vaping human adult. We first generated aerosols from 50:50 PG:VG e-liquid supplemented with menthol flavoring only. This allowed us to eliminate possible confounding influence by other (often non-disclosed or proprietary) chemicals found in commercially available ECs. We discovered that addition of menthol to PG:VG even at the very low dose of 0.1% leads to enhanced particle counts in all tested size fractions (300 nm–10 µm) (Fig. 1), similar to the effect of VEA we previously reported [16], given its strong link with the EVALI [12, 13]. The impact of menthol on sub-micron particle count increased dose-dependently as the concentration of menthol in the e-liquid base went up to 8%. Nevertheless, despite significantly higher amounts of inhaled particles (for all size fractions) at 8% vs. 0% and 0.1% menthol, no significant difference between 1 and 8% on quantity of 1–10 µm particles was evident (Fig. 1). We chose 0–8% v/v (equating to 0–80 mg/mL) range for menthol concentration as a representative window for evaluation based on prior publications [23–26]. We also analyzed seven commercially available e-liquids (Table 1) and found 0.4–4.1% as the concertation range for menthol.

Next, we investigated whether the impact of menthol on inhaled particle count is also evident in marketed EC products. Thus, we applied HUMITIPAA and studied the highly popular off-the-shelf menthol-flavored Vuse Alto along with its non-menthol (tobacco-) flavored counterpart (Vuse Alto Golden Tobacco) (Fig. 2). Prior to inhaled particle characterization, we analyzed the two Vuse e-liquids for their chemical composition by GC–MS (Table 2). The PG:VG ratio was 38:52 and 41:53 in Vuse Alto Menthol and Vuse Alto Golden Tobacco, respectively. Thus, in our particle analysis (Fig. 2) to ensure that such difference in PG and VG does not confound interpretation of the findings, we compared results against freshly prepared e-liquid bases reconstituted with corresponding PG and VG contents—that is PG:VG ratios of 38:62 (hereafter referred to as 38% PG; that is PG control for Vuse Alto Menthol), 41:59 (hereafter referred to as 41% PG; that is PG control for Vuse Alto Golden Tobacco), 47:53 (hereafter referred to as 53% VG; that is VG control for Vuse Alto Golden Tobacco), and 48:52 (hereafter referred to as 52% VG; that is VG control for Vuse Alto Menthol). As shown in Fig. 2, we observed that Vuse Menthol compared with Vuse Golden Tobacco, 38% PG and 52% VG (its matching PG and VG content controls) as well as 50:50 PG:VG e-liquids generates significantly higher quantities of 1–2.5 μm, 2.5–4 μm, and 4–10 μm particles upon aerosolization (Fig. 2, top right and bottom panels). For 300 nm–1 μm size fraction, the quantity of aerosols emitted from Vuse Menthol did not differ significantly compared with other conditions (Fig. 2, top left panel). Interestingly, the number of sub-micron particles generated from Vuse Golden Tobacco EC was significantly lower compared with its 50:50 PG:VG. Nevertheless, there was no statistically significant difference in 300 nm–1 μm particles between Vuse Golden Tobacco condition and Vuse Menthol EC, or its matching PG and VG content control e-liquids (41% PG and 53% VG) (Fig. 2, top left panel). Notably, the GC–MS-measured concentration of nicotine in both Vuse products was about 6% which is considerably higher than advertised 1.8% strength (Table 2).

**Table 2:** Gas chromatography–mass spectroscopy analysis of vuse alto menthol and vuse alto golden tobacco pods e-liquid

Vuse alto menthol pod (sold as 1.8% nicotine strength)		Vuse alto golden tobacco pod (sold as 1.8% nicotine strength)	
Analyte	Abundance (%)	Analyte	Abundance (%)
Glycerin	51.6	Glycerin	52.9
Propylene glycol	38.0	Propylene glycol	40.7
Nicotine	5.9	Nicotine	6.1
Menthol	3.8	Trimethyl borate	0.3
Unknown	0.7	Unknown	0.1
Trimethyl borate	0.1		

**Fig. 3:**
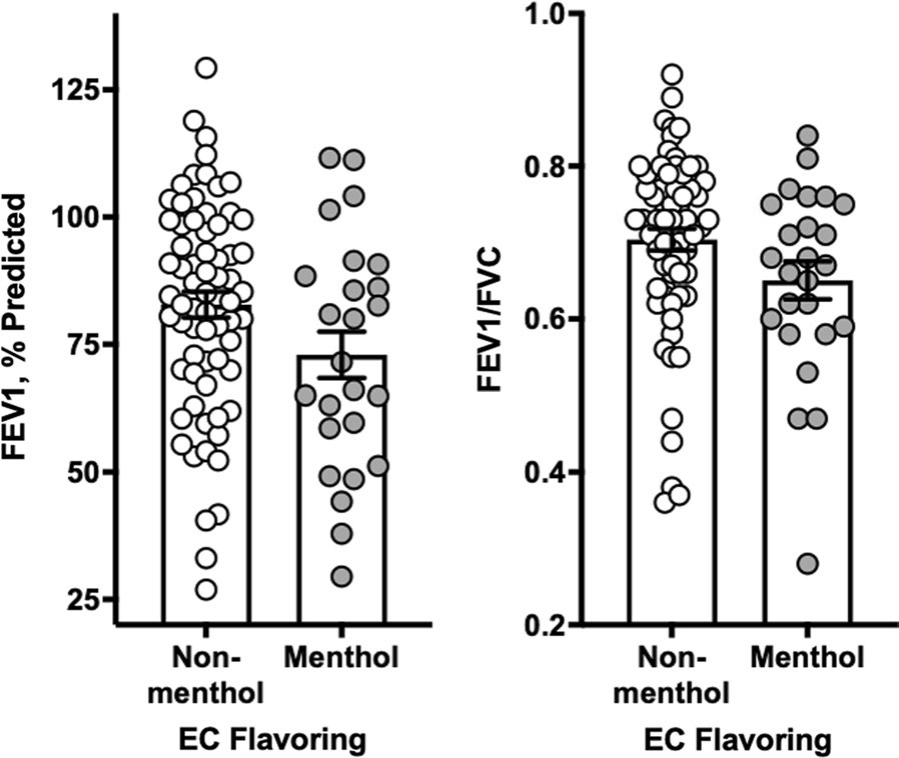
Association Menthol Flavoring in Electronic Cigarettes with Lung Function. The mean FEV1 was 9.9% predicted lower (95% CI 0.1, − 19.8% pred; *P* = 0.053) while the mean FEV1/FVC was 0.06 lower (95% CI 0.002, − 0.11; *P* = 0.058) in an unadjusted comparison of 25 users of menthol-flavored ECs with 69 users of other flavored ECs in the COPDGene cohort (see Table 3). These associations persisted after adjustment for age, gender, race, pack years of smoking, as well as the use of nicotine or cannabis-containing vaping products in multivariate models. Specifically, menthol users had on average 9.6% predicted lower FEV1 (95% CI − 0.2, − 19.1% pred; *P* = 0.04) and 0.06 lower FEV1/FVC (95% CI − 0.01, − 0.12; *P* = 0.01) compared to users of other flavored products adjusted for these covariates. *FEV1* Forced Expiratory Volume in the First Second, *FVC* Forced Vital Capacity, *COPDGene* Chronic Obstructive Lung Disease Gene study

We then sought evidence of toxicity of menthol-flavored electronic cigarettes in a human cohort to complement our preclinical findings. The cohort with the largest number of EC users we could identify was COPDGene. Our COPDGene clinical data analysis included 94 individuals: 25 menthol vapers and 69 users of other flavored vaping products. The characteristics of these individuals are compared in Table 3. Menthol users had lower average age and were more frequently women and African American, although these differences were not statistically significant (all *P* > 0.05). The tobacco exposure history was quite similar between the two groups, including pack years, duration of tobacco smoking, and average cigarettes/day. Users of non-menthol flavored products reported more frequent use of cannabis and less frequent use of nicotine. FEV1, % predicted, and FEV1/FVC were lower in menthol users with a borderline *p*-value. An association between menthol use and reduced lung function was present after adjustment for age, gender, race, pack-years of smoking, and the use of nicotine or cannabis-containing vaping products. Specifically, menthol users had, on average, 9.6% predicted lower FEV1 compared to users of other flavored products adjusted for these covariates (95% CI − 0.2, − 19.1% pred; *P* = 0.04) (Fig. 3). Similarly, menthol users had, on average, 0.06 lower FEV1/FVC compared to users of other flavored products adjusted for the same covariates (95% CI − 0.01, − 0.12; *P* = 0.01) (Fig. 3).

**Table 3:** Comparison of users of menthol vs. non-menthol flavored electronic cigarettes in COPDGene study

	Total	Non-menthol flavors	Menthol	***P***
N (sample size)	94	69	25	
Age, years	65.2 (6.3)	65.6 (6.6)	64.1 (5.5)	0.32
Gender, %				0.58
Male	44.7%	46.4%	40.0%	
Female	55.3%	53.6%	60.0%	
Race, %				0.38
Caucasian	67.0%	69.6%	60.0%	
African American	33.0%	30.4%	40.0%	
Current tobacco smoking, %	56.4%	53.6%	64.0%	0.37
Years since quitting tobacco	4.0 (2.0–6.0)	4.0 (1.5–6.0)	5.0 (4.0–7.0)	0.37
Pack-years of tobacco smoking	43.3 (30.8–57.5)	43.4 (30.8–55.8)	43.2 (31.2–57.5)	0.79
Duration of smoking tobacco, years	46.0 (42.0–50.7)	47.0 (42.4–51.0)	45.0 (41.0–48.2)	0.33
Average cigarettes/day, last 5 years	10.0 (8.0–20.0)	10.0 (8.0–20.0)	11.0 (6.0–20.0)	0.79
Resting oxygen saturation, %	96.3 (2.5)	96.2 (2.5)	96.6 (2.5)	0.58
Body mass index, kg/m^2^	29.2 (6.7)	29.2 (6.8)	29.1 (6.5)	0.93
MMRC dyspnea score				0.42
0	53.2%	49.3%	64.0%	
1	6.4%	7.2%	4.0%	
2	13.8%	17.4%	4.0%	
3	21.3%	21.7%	20.0%	
4	5.3%	4.3%	8.0%	
St George's Respiratory Questionnaire	19.2 (7.2–43.0)	18.4 (9.8–41.6)	19.9 (6.5–49.1)	0.86
Duration of e-cig use, months	12 (22)	12 (20)	12 (34)	0.62
E-cig contains nicotine	71.3%	66.7%	84.0%	0.10
E-cig contains cannabis	12.8%	17.4%	0.0%	0.03
FEV1, % predicted	80.1 (21.7)	82.8 (20.9)	72.9 (22.7)	0.052
FEV1/FVC	0.69 (0.12)	0.70 (0.12)	0.65 (0.12)	0.058
Bronchodilator response, %	13.8%	15.9%	8.0%	0.30
Spirometric GOLD stage				0.46
PRISM	14.9%	13.0%	20.0%	
GOLD 0	54.3%	56.5%	48.0%	
GOLD 1	5.3%	2.9%	12.0%	
GOLD 2	16.0%	15.9%	16.0%	
GOLD 3	5.3%	5.8%	4.0%	
Percent emphysema (LAA% − 950)	2.2 (0.8–5.6)	2.4 (0.8–5.6)	1.6 (0.8–5.4)	0.62
% Gas trapping	12.7 (5.1–30.0)	11.1 (5.0–27.9)	19.5 (10.4–35.0)	0.27

*MMRC* Modified Medical Research Council, *GOLD *Global Initiative for Obstructive Lung Disease, *PRISM* Preserved Ratio Impaired Spirometry, *LAA%* % of Low Attenuation Areas in Density Mask Analyses of Chest CT Scans

*P* values listed in the last column compare users of menthol flavors with users of non-menthol flavoring.

## Discussion

The similarity of 1–10 µm particle profiles between the content-controlled e-liquids (Fig. 1)—i.e., 50:50 PG:VG mixtures supplemented with menthol flavoring, and the commercial menthol-flavored off-the-shelf (Vuse) product we tested (Fig. 2) support our hypothesis that menthol flavoring enhances particle quantities in aerosols from ECs. Testing e-liquids with PG (38%, 41%) and VG (52%, 53%) levels other than 50% that we used as our basic control but instead matched the products’ abundance levels (Fig. 2) confirmed that such difference in particle count was not necessarily due to altered PG, VG in e-liquid. In addition, including a non-menthol flavored EC allowed us to further demonstrate role of menthol in increasing inhaled particle quantities. On the other hand, it was unexpected that such effect was not evident for 300 nm–1 µm size fraction. This can, however, be potentially attributed to presence of unknown and known compounds (e.g., trimethyl borate) in the Vuse e-liquid (Table 2). Additionally, we previously demonstrated inhibitory effect of nicotine (tested at 0.6–2.4% doses) on inhaled particle count from ECs [16]. As such, we speculate that unexpectedly higher content of nicotine in Vuse pods (~ 6%) may contribute to this effect and mask menthol-driven increase in particle generation.

Enhanced particle count with vaping is important in the context of both EC only users and combustion cigarette smokers. Increase in the number of particles will increase the mass of EC aerosols delivered to the lung. EC aerosols are known to contain many harmful substances such as nicotine and multifunctional carbonyls, including formaldehyde. A number of in vitro and in vivo studies suggest that EC aerosols can provoke lung inflammation, oxidative stress, DNA damage, airway hyperresponsiveness, induce epithelial-to-mesenchymal transition, impact cellular viability, and impair lung function and anti-pathogen immune responses [27–35]. Therefore, increased aerosolization of EC aerosol quantity due to addition of menthol flavoring would be expected to have multiple harmful consequences for the lungs.

A major limitation of our pre-clinical study is the lack of analysis of particles < 300 nm. Currently we have been unable to integrate any commercially available nano-sensor into the HUMITIPAA mostly due to their inability for real-time analysis. Thus, we are designing and developing new sensors for future use with our robotic system. However, this would not impact clinical relevance of our findings as the particle range we analyzed remains important to the study of inhaled particles from vaping products and during environmental exposures. Another limitation of our experimental studies is focus on physical characteristics of inhaled particles only. The chemical composition of the particles following menthol flavoring addition can also play an important role in subsequent pulmonary toxicity. The real-time chemical analysis of aerosols and vapors from EC emissions can be a possibility by infrared spectroscopy. However, integration of this approach with HUMITIPAA is complex and requires extensive engineering of our system to ensure compatibility and appropriate communication between all system components. For this reason, we have initiated development of an add-on module to allow coupling physical–chemical characterizations of the airborne particles in future. Additionally, we plan in follow-up studies to link the HUMITIPAA with lung Airway-on-a-Chip biodevices that we have previously developed [36–38] to directly compare toxicological impact of inhaled particles form ECs + /– menthol flavoring on viability and functionality of cells and tissues present in human conducting airways. This would also enable us to take into consideration impact of non-particulate gaseous component of the EC emissions.

Our clinical study has several limitations. First, our sample size was modest. 10-year follow-up visits in the COPDGene cohort are ongoing; therefore, we hope to repeat our analysis with a larger sample size soon. Second, all participants were either current or former smokers with and without spirometry airflow obstruction. We thoroughly investigated differences in tobacco exposure between menthol and non-menthol users, including current smoking, pack-years of smoking, duration of smoking, years since quitting, and the number of cigarettes smoked per day (Table 3). None of these parameters were different, which reassured us that tobacco exposure was unlikely to be a major confounder of the relationship between menthol use and impaired lung function. However, we could not adjust for the use of menthol-flavored combustion cigarettes as data on the same was unavailable for Phase 3 visits in COPDGene. Therefore, it is possible that the worse lung function noted in users of menthol-flavored e-cigarettes was due to more frequent use of menthol-flavored combustion cigarettes, i.e., dual menthol use, compared to users of non-menthol-flavored e-cigarettes. Third, the duration of EC use was modest. This is a challenge for most clinical EC research studies as the widespread use of ECs is a recent phenomenon. Nonetheless, we used data from the 10-year rather than 5-year COPDGene study visit as the duration of EC use was longer. Also, data on EC use was collected at 5-year intervals; therefore, we lacked granular information on the consistency of the use of flavoring compounds over time. Finally, the use of menthol flavoring was more prevalent in African Americans, who also have lower lung function than Caucasians. Accordingly, we used race-specific prediction equations for our lung function data and adjusted for race in our multivariate model. Some have suggested that race-specific prediction equations should not be used [39]. Had we not used race specific equations, the differences in lung function between menthol users vs. users of other flavored e-cigarettes would have been even larger as there were more African Americans among the menthol group.

Altogether, our findings provide new evidence of an association with altered lung function and increased number of particles inhaled due to menthol chemical addition to e-liquid. Moreover, it sets a foundation for future large-scale prospective clinical studies to demonstrate a robust relation.

## Conclusions

Here we merged biomimetic robotic engineering and analysis of a large clinical dataset to uncover evidence for a negative impact of menthol-flavored ECs on lung function and inhaled particle quantity. The epidemiological association identified between the use of menthol-flavored products and the reduced lung function complemented our in vitro findings. Additionally, our results demonstrate utility of the HUMITIPAA as a predictive technology to identify pulmonary toxicity potential of ECs when the chemical formulation of e-liquid has been modified, for instance when a given flavoring has been added. This is in line with our earlier observation where we identified significantly higher quantities of inhaled particles when VEA was present in the EC liquid [16]; and this in vitro finding was in line with the reported clinical outcome (enhanced morbidity and mortality) among VEA-containing vaping device users.

## Supplementary Information

**Additional file 1:** **Table S1**. Summary of PG and VG Volumes Used to Create PG:VG Controls for Vuse E-liquid in Studies in Fig. 2.

## Data Availability

The datasets used and/or analyzed during the current study are available from the corresponding author on reasonable request.

## Abbreviations

EC: Electronic cigarette
HUMITIPAA: Human vaping mimetic real-time particle analyzer
COPDGene: Chronic Obstructive Lung Disease Gene Study
FEV1: Forced expiratory volume in the first second
FVC: Forced vital capacity
VEA: Vitamin E acetate
PG: Propylene glycol
VG: Vegetable glycerin
ISO: International Organization for Standardization
GC–MS: Gas chromatography–mass spectroscopy
LAA: % Of low attenuation areas in density mask analyses of chest CT scans
MMRC: Modified Medical Research Council
GOLD: Global initiative for obstructive lung disease
PRISM: Preserved ratio impaired spirometry
