# Supplementary material for: Electronic cigarette menthol flavoring is associated with increased inhaled micro and sub-micron particles and worse lung function in combustion cigarette smokers
Source: Respir Res. 2023 Apr 11;24:108. doi: 10.1186/s12931-023-02410-9 (PMC10088218; doi:10.1186/s12931-023-02410-9)
Supplement: Supplementary file 1 — Additional file 1: Table S1. Summary of PG and VG Volumes Used to Create PG:VG Controls for Vuse E-liquid in Studies in Fig. 2. [file 12931_2023_2410_MOESM1_ESM.pdf]

Additional file 1

Electronic Cigarette Menthol Flavoring is Associated with Increased Inhaled Micro and Sub-micron Particles and Worse Lung Function in Combustion Cigarette Smokers

AUTHORS

Divay Chandra[1#], Rachel F. Bogdanoff[1#], Russell P. Bowler[2], Kambez H. Benam[1,3,4*]

AFFILIATIONS

[1]Division of Pulmonary, Allergy and Critical Care Medicine, Department of Medicine, University of Pittsburgh, Pittsburgh, PA 15213, USA.

[2]Division of Pulmonary, Critical Care and Sleep Medicine, National Jewish Health, Denver, CO 80206, USA.

[3]Department of Bioengineering, University of Pittsburgh, Pittsburgh, PA 15219, USA.

[4]Vascular Medicine Institute, University of Pittsburgh, Pittsburgh, PA 15213, USA.

[#]: These authors contributed equally.

CONTACT

*Corresponding author: benamk@pitt.edu

PG:VG Ratio	50:50 PG:VG (mL)	VG (mL)
**38:62**	19.0	6.0
**41:59**	20.5	4.5
**47:53**	23.5	1.5
**48:52**	24.0	1.0
**50:50**	25.0	0

Table S1. Summary of PG and VG Volumes Used to Create PG:VG Controls for Vuse E-liquid in Studies in Fig. 2.
